# Temperature-Based Long-Term Stabilization of Photoacoustic Gas Sensors Using Machine Learning

**DOI:** 10.3390/s24237518

**Published:** 2024-11-25

**Authors:** Pavel Borozdin, Evgenii Erushin, Artem Kozmin, Anastasia Bednyakova, Ilya Miroshnichenko, Nadezhda Kostyukova, Andrey Boyko, Alexey Redyuk

**Affiliations:** 1 The Artificial Intelligence Research Center, Novosibirsk State University, Pirogova Str. 2, 630090 Novosibirsk, Russia; 2 Faculty of Physical Engineering, Novosibirsk State Technical University, 20 Prospekt K. Marksa, 630073 Novosibirsk, Russia

**Keywords:** photoacoustic gas sensor, photoacoustic spectroscopy, optical sensing, methane, long short-term memory networks, machine learning, neural networks, sensitivity enhancement, accuracy

## Abstract

In this study, we address the challenge of estimating the resonance frequency of a photoacoustic detector (PAD) gas cell under varying temperature conditions, which is crucial for improving the accuracy of gas concentration measurements. We introduce a novel approach that uses a long short-term memory network and a self-attention mechanism to model resonance frequency shifts based on temperature data. To investigate the impact of the gas mixture temperature on the resonance frequency, we modified the PAD to include an internal temperature sensor. Our experiments involved multiple heating and cooling cycles with varying methane concentrations, resulting in a comprehensive dataset of temperature and resonance frequency measurements. The proposed models were trained and validated on this dataset, and the results demonstrate real-time prediction capabilities with a mean absolute error of less than 1 Hz for frequency shifts exceeding 30 Hz over four-hour periods. This approach allows continuous, real-time tracking of the resonance frequency without interrupting the laser operation, significantly enhancing gas concentration measurements and contributing to the long-term stabilization of the sensor. The results suggest that the proposed approach is effective in managing temperature-induced frequency shifts, making it a valuable tool for improving the accuracy and stability of gas sensors in practical applications.

## 1. Introduction

Accurately determining the concentration of various gases in the environment is a crucial task in industry, scientific research, and safety sectors. For instance, monitoring the concentration of explosive gases like hydrocarbons helps prevent accidents in coal mines [1] and industrial plants, while measuring oxygen [2] and carbon dioxide [3] levels in the atmosphere is critical for the aerospace industry. Gas sensors play a key role in these processes, ensuring safety and controlling air quality in diverse applications. These sensors are widely used in the oil, gas [4], and chemical industries for monitoring air pollution in urban and industrial areas [5], in medicine [6], and in many other fields [7,8].

Gas sensors can be classified into four main types: analytical, electrochemical, semiconductor, and laser absorption-based. Among these, laser absorption spectroscopy-based analyzers (such as photoacoustic sensors) offer several distinct advantages, including short response times, low detection limits reaching parts per billion (ppb), and the capability for real-time measurements [9,10,11]. The operating principle of these sensors was first described in [12]. When a gas absorbs radiation, it heats up, causing the pressure to rise. If the light is modulated at an acoustic frequency, the resulting rapid pressure changes generate sound waves. The frequency of these pressure fluctuations matches the modulation frequency of the light, and the intensity of the sound increases with the concentration of the target gas.

A key requirement for measuring the gas concentration is the accurate determination of the gas cell’s resonance frequency, which can change over time. In a typical setup, a speaker is activated at system startup to induce oscillations within the acoustic gas cell. An analog-to-digital converter (ADC) measures the resulting sound signal, performs a Fourier transform, and identifies the resonance frequency within a specified range. Once this frequency is determined, the readings from the differential amplifier, $U_{micro}$, and the laser output power, $U_{laser}$, are recorded simultaneously during the experiment. These readings constitute the “raw” data used to calculate the gas concentration. In our previous work [13], we described in detail the procedure for processing raw data to determine the methane concentration.

Currently, there are two well-established approaches for determining the resonance frequency of a gas cell. The first method involves using a speaker to identify the resonance frequency [13,14], while the second relies on adjusting the laser modulation to find the system’s maximum response [9,15]. However, both methods share the limitation of interrupting gas concentration measurements. For example, under fluctuating temperature and concentration conditions, these algorithms provide only short-term accuracy. Consequently, the resonance frequency must be remeasured, resulting in a pause in gas concentration measurements. Under typical conditions for an ideal gas, in our experimental setup, a 1 °C change in gas temperature can locally shift the resonance frequency by approximately 3 Hz, significantly impacting the accuracy of the gas concentration determination. In our earlier experiments involving prolonged concentration measurements, we observed that the measurement error increases by more than 20% within 7–10 min of continuous measurement without changing the external conditions. The high-quality factor of the acoustic resonator leads to a rapid shift in the resonance frequency due to heating, significantly impacting the measurement accuracy. In [16], it was shown that the photoacoustic gas cell’s resonance frequency shifts by 47 Hz when the temperature changes from 20 °C to 50 °C.

The study presented in [17] addressed this issue by employing an extremum-seeking control algorithm. This approach introduces sinusoidal modulation to the laser pulse frequency, allowing the system to determine the frequency that produces the maximum photoacoustic detection (PAD) signal. Physically, this peak PAD signal is achieved when the pulse repetition frequency aligns with the cell’s resonance frequency. However, the method has a drawback: the parameters of the sinusoidal modulation must be precisely tuned for each specific experimental setup to ensure accurate results. This requirement for precise parameter adjustment presents a separate challenge, as configurations optimized for one set of conditions may not be applicable to others.

In this work, we investigate both theoretically and experimentally how changes in temperature and gas concentration affect resonance frequency shifts, demonstrating that temperature variations are the primary driver of resonance frequency drift. To address this, we propose an alternative solution for determining the resonance frequency under fluctuating cell temperatures: predicting the cell resonance frequency based on its temperature data. This approach allows for real-time tracking of the resonance frequency without interrupting laser operation, enabling the system to dynamically adjust the laser to the correct frequency. To implement this solution, we enhanced our experimental setup by integrating a temperature sensor within the cell. We developed several models to predict the cell resonance frequency based on collected temperature data, utilizing both physical principles and advanced neural network (NN) techniques with different architectures. Our neural network models are built on long short-term memory (LSTM) networks, which are well suited for processing time-series data [18,19], as well as a self-attention mechanism [20,21], which allows a model to learn long-range dependencies in the input data.

The main contributions of this study are as follows: (i) We develop a novel gas cell design incorporating an embedded temperature sensor for more precise control of the gas mixture temperature and its influence on the resonance frequency; (ii) We develop and implement an approach using LSTM networks to predict the resonance frequency from temperature data, effectively capturing complex temporal patterns; (iii) We achieve significant improvements in the long-term stability and accuracy of the sensor, enabling continuous and stable operation over extended periods without the need to interrupt measurements.

The rest of the paper is organized as follows. Section 2 describes the developed experimental setup. In Section 3, we present several theoretical approaches for describing the gas state and key equations linking the cell resonance frequency to temperature. Section 4 details the collected experimental data and its processing. In Section 5, we outline our models for restoring the resonance frequency based on temperature and discuss the results of their training. Finally, a discussion of our findings and the conclusions are provided in Section 6 and Section 7, respectively.

## 2. Experimental Setup

**Figure 1:**
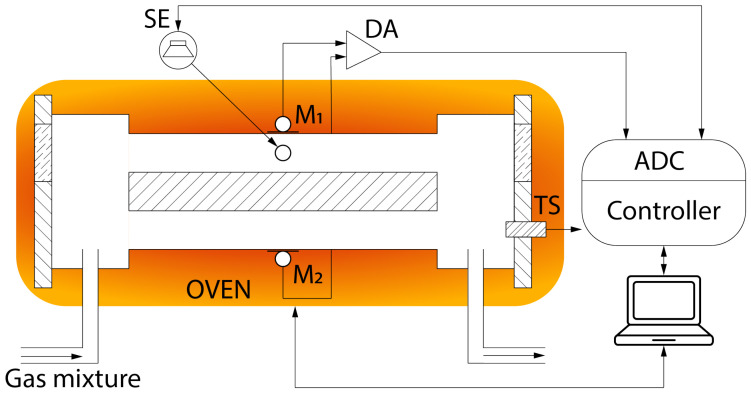
Scheme of the experimental setup: SE—sound emitter; DA—differential amplifier; M_1_, M_2_—microphones; TS—temperature sensor; ADC—analog-to-digital converter.

The experimental setup is illustrated in Figure 1. The measurement cell is a resonant differential photoacoustic detector, consisting of two cylindrical acoustic channels, each measuring 90 mm in length and 9 mm in diameter. These channels are aligned parallel to each other and separated by a partition. Additionally, buffer cavities measuring 8 mm in length and 20 mm in diameter are incorporated, enclosed by flanges fitted with anti-reflective-coated Zinc Selenide windows. Gas is introduced into the measuring system through 2 mm internal diameter hoses mounted on the walls of the buffer cavity, and the PAD is filled with a test gas mixture to achieve atmospheric pressure before sealing.

The detector is equipped with two CME-1538-100LB electret microphones (CUI devices), each with a diameter of 4 mm, denoted as M_1_ and M_2_, which are positioned along the central axis of the acoustic channels. For precise signal balancing, each microphone is individually connected to the differential amplifier (DA), with one channel capturing the laser interaction with the test gas and the other serving as a reference to cancel noise using the DA.

To determine the fundamental resonant frequency, a small piezoelectric sound emitter (SE) is placed along the central axis of one of the acoustic channels, directly opposite the microphone. The method for determining the PAD’s resonant frequency with an error margin of around ±0.1 Hz is outlined in [13,14]. The minimum resonant frequencies observed are approximately 1750 Hz in air and 1780 Hz in nitrogen, with a Q-factor of ≈40 for the detector used. Signals from both the DA and the SE are transmitted to an analog-to-digital converter (ADC) of the controller, which is connected to a personal computer (PC).

It is important to note that external vibrations can considerably affect the precision of resonant frequency detection. In our experiment, we took measures to reduce the effect of external vibrations by placing the setup on a vibration-isolating surface. Measurements were conducted in a stable laboratory environment to minimize external noise and oscillations, thus enhancing the accuracy of resonance frequency estimation. Although this study does not specifically analyze the influence of vibrations, a future investigation is planned to address this aspect.

To investigate the effect of the gas mixture temperature on the resonant frequency, the PAD used in earlier studies [13,17] was modified to incorporate a temperature-resistive platinum sensor (TS). The sensor is mounted on a printed circuit board, with a corresponding hole drilled in the buffer cavity of the PAD, sealed using a rubber gasket. Signals from the TS are transmitted to the controller, which then sends the data to the PC for further processing. To regulate the temperature of the measurement cell, we employed an oven with a Peltier element, managed by an MTDEVAL1 Thorlabs controller.

## 3. Theory

### 3.1. Gas Models

To understand how the resonance frequency of a gas cell is influenced by temperature, we consider several models that describe the state of the gas. In the ideal gas model, the equation of state is given by

(1) PV=νRT,

where *P* represents the pressure, *V* is the volume, ν is the amount of substance, *R* is the universal gas constant, and *T* is the gas temperature. As laser radiation passes through the cell, the gas molecules absorb this radiation, resulting in an increase in temperature and, consequently, pressure, as described by Equation (1). When the laser pulse is interrupted, the gas pressure decreases, causing pressure oscillations—essentially, sound waves. The resonance frequency of the cell is determined by these sound oscillations, which depend on both the cell’s dimensions and the speed of sound in the gas. Therefore, to understand how the resonance frequency varies with temperature, we must first establish the relationship between the speed of sound in the gas and its temperature. For an ideal gas, the speed of sound is given by

(2) $${\hat{c}}_{s}=\sqrt{\frac{\gamma RT}{M}},$$

where $\gamma =c_{P}/c_{V}$ is the adiabatic index and *M* is the molar mass of the gas.

To extend the ideal gas equation of state (1) to account for real gas behavior, we use the form $PV=\nu RT(1+\frac{B(T)\nu }{V}+\cdots )$ [22]. Consequently, the speed of sound in a real gas, based on Equation (2), can be expressed as

(3) $$c_{s}=\sqrt{\frac{\gamma (RT+2PB(T))}{M}}={\hat{c}}_{s}\cdot \sqrt{1+\frac{2\nu }{V}B\left(T\right)\left(1+\frac{\nu }{V}B\left(T\right)\right)}={\hat{c}}_{s}\cdot \kappa ,$$

where κ is a coefficient that depends on the specific properties of the gas. If the gas is confined within a cell of an effective length *L*, the resonance frequency of the cell, using Equation (3), is approximately

(4) $$f^{r}\approx \frac{c_{s}}{L}=\frac{\kappa }{L}\cdot {\hat{c}}_{s}.$$

By substituting Equation (2) and assuming that the temperature dependence of κ and γ is negligible, we can derive the proportional relationship between the resonance frequency and the gas temperature *T* (in Kelvin) (see Appendix A):

(5) $$f^{r}\propto \sqrt{T}.$$

For a multicomponent gas mixture, the adiabatic index and the molar mass of the mixture are calculated as follows:

(6) $$\gamma \left(T,\vec{n}\right)=1+\frac{1}{\sum \frac{n_{i}}{\gamma_{i}\left(T\right)-1}}\approx 1+\frac{1}{\sum \frac{n_{i}}{\gamma_{i}-1}},$$

(7) $$M\left(\vec{n}\right)=\sum m_{i}n_{i}=\left({\vec{m}}^{T}\cdot \vec{n}\right),\;\;\sum n_{i}=1,$$

where $\vec{n}={\left(n_{1},\cdots ,n_{k}\right)}^{T}$ represents the concentration vector of the gas components and $\vec{m}={\left(m_{1},\cdots ,m_{k}\right)}^{T}$ is the vector of their respective molar masses. Therefore, in a multicomponent mixture, the resonance frequency depends not only on the temperature but also on the concentrations of the mixture components, i.e., $f^{r}=f^{r}\left(T,\vec{n}\right)$.

As demonstrated in Appendix A, at certain concentrations of an impurity gas, the shift in the resonance frequency caused by temperature changes is significantly larger than the shift due to variations in the concentrations of the mixture components ($\Delta f_{\mathrm{ppm}}^{r}/\Delta f_{K}^{r}$ = $10^{-4}$ from pure $N_{2}$ to 1% ${\mathrm{CH}}_{4}$ in $N_{2}$, see Table A3). Therefore, when measuring methane concentrations below 1% (10,000 ppm), it can be reasonably assumed that the entire shift in the resonance frequency is attributable to changes in the gas temperature (see Table A3). For sulfur hexafluoride, this assumption holds true only for concentrations below 0.1% (1000 ppm).

For most practical applications, a methane concentration measurement with an upper limit of 1% is sufficient. This suggests the feasibility of developing a model to determine the cell’s resonance frequency based solely on the gas temperature, enabling continuous concentration measurements without the need for a speaker inside the resonator. Removing the speaker from the resonator enhances the resonance characteristics and improves the accuracy of gas concentration measurements.

### 3.2. Heat Equation

The distribution of temperature within the gas and its dependence on various factors for concentration measurements are governed by the heat conduction equation:

(8) $$\frac{\partial u}{\partial t}-\alpha^{2}\;△u=f\left(\vec{r},t\right),\;\;\alpha^{2}=\frac{\kappa }{c_{P}\rho },$$

where the function $u(\vec{r},t)$ represents the temperature at position $\vec{r}$ and time *t*, $△=\nabla^{2}$ is the Laplace operator, $f(\vec{r},t)$ describes the heat sources present in the system, and $\alpha^{2}$ is the thermal diffusivity coefficient.

Accurately determining the average gas temperature in real time is challenging due to the lag between the heating of the temperature sensor and the gas itself. To address this, solving the differential heat conduction Equation (8) is essential for the precise estimation of both the gas temperature and the corresponding resonance frequency of the cell. However, solving this equation requires complete knowledge of the heat sources and the physical properties of both the gas and the cell. Any inaccuracies in estimating the actual gas temperature will directly affect the accuracy of the resonance frequency determination. To overcome these challenges, we propose an approach based on LSTM neural networks, which effectively resolves these issues and provides high-accuracy resonance frequency predictions without requiring detailed knowledge of the system’s physical parameters.

## 4. Data Collection and Processing

The experimental dataset consists of measurements obtained from multiple experiments, during which both the temperature and resonant frequency of a gas cell containing various gas mixtures were recorded. The gas mixtures investigated include $N_{2}$ with 1010 ppm of ${\mathrm{CH}}_{4}$, $N_{2}$ with 97 ppm of ${\mathrm{CH}}_{4}$, $N_{2}$ with 9.7 ppm of ${\mathrm{CH}}_{4}$, and $N_{2}$ with 2 ppm of ${\mathrm{CH}}_{4}$.

**Figure 2:**
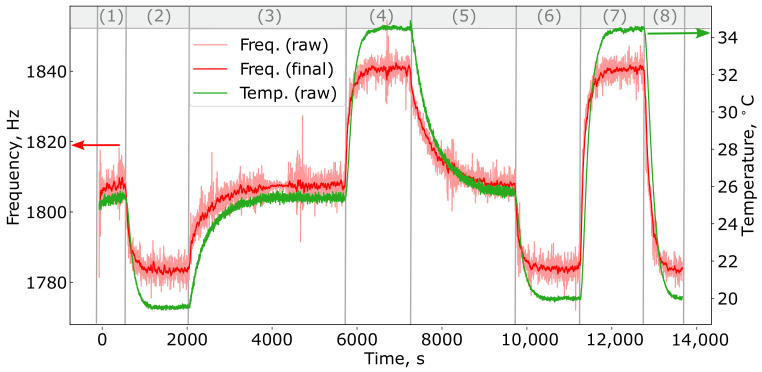
Time series of the resonance frequency $f_{t}^{r}$, shown both in its original form (raw, pink curve) and after applying smoothing (final, red curve), along with the original time series of the temperature $T_{t}$ (raw, green curve).

The experiments consisted of multiple heating and cooling cycles of the PAD, utilizing various temperature gradients. Figure 2 presents the experimental data for a gas concentration of 1010 ppm of methane. Each cycle started with a 10-minute measurement period at room temperature (plateau phase, indicated as zone (1) in Figure 2), during which the gas cell was allowed to remain undisturbed. Subsequently, the cell was cooled to 20 °C using a thermostat based on a Peltier element (zone (2)). After reaching this temperature, the cell naturally warmed back to room temperature, approximately 26.5 °C, through heat exchange with the environment (zone (3)). The Peltier element was then used to heat the cell to around 35 °C (zone (4)), followed by another natural cooling period back to room temperature (zone (5)). This process was repeated, with the cell being cooled again to about 20 °C (zone (6)), reheated to around 35 °C (zone (7)), and finally cooled back to 20 °C (zone (8)). After each heating or cooling step, the cell was held at a steady-state temperature for approximately 10 minutes.

During the experiments, temperature and resonant frequency measurements were recorded every 2 seconds. The temperature was measured using a resistive platinum Pt1000 sensor with an accuracy of 0.1 °C, while the resonant frequency was captured with built-in microphones and an emitter. This experimental setup generated two synchronized time series: one representing the temperature and the other the resonant frequency, both sampled at the same 2-second intervals.

We preprocessed the experimental data by smoothing the resonant frequency to minimize outliers and reduce the magnitude of fluctuations. For this, we applied the Simple Moving Average (SMA) algorithm, following the approach detailed in [23]. Figure 2 presents an example of the resonance frequency time series before smoothing (red curve) and after applying the SMA algorithm (black curve). The time-series data used to train our models included the preprocessed resonance frequency, denoted as $f_{t}^{r}$, and the temperature of the gas cell, $T_{t}$.

Additionally, the temperature time series $T_{t}$ was differentiated with respect to time to compute the temperature derivative $\frac{\partial T}{\partial t}\left(t\right)$ for each experiment. This derivative was used as an additional feature for model training. To calculate the temperature derivative $\frac{\partial T}{\partial t}\left(t_{0}\right)$ at a given time $t_{0}$, we fitted the experimental temperature data to a polynomial and computed the left-hand derivative of that polynomial at $t=t_{0}$, following the methods outlined in [24,25].

After preprocessing, the data from each experiment at various concentrations were randomly split into training, validation, and test subsets in proportions of 70/15/15%. To enable model training on data from multiple concentrations, the subsets for all concentrations were merged into a single combined dataset. To ensure the combined dataset was of comparable size to the individual concentration subsets, a portion of data points was randomly excluded. Additionally, a full dataset containing all concentration measurements, without any data reduction, was created. This larger dataset, four times the size of the individual concentration sets, allowed for model training on the entire range of available data.

Table 1 presents an overview of the datasets used in this study, including the number of data points for each set. The table also provides the temperature range of the gas cell, $T_{t}$, and the corresponding resonance frequency range, $f_{t}^{r}$, for each dataset.

## 5. Description of the Models

In this section, we provide an overview of the models developed to predict the resonance frequency of the gas cell based on temperature data. First, in Section 5.1, we discuss the results obtained from a basic theoretical model that establishes a fundamental relationship between temperature and resonance frequency. Then, in Section 5.2, we introduce advanced models based on recurrent neural networks, specifically LSTM networks, which are designed to improve the accuracy and reliability of resonance frequency predictions.

### 5.1. Simple Theoretical Model

According to the basic theoretical model (5), the square of the resonance frequency is assumed to have a linear relationship with the gas temperature (in Kelvin). The fitting process was performed using the least-squares method, following the equation

(9) $${\left(f_{t}^{r}\right)}^{2}=\alpha_{0}+\alpha_{1}\cdot T_{t},$$

where $\alpha_{0}$ and $\alpha_{1}$ are free parameters. The fitting procedure was carried out using the open-source SciPy library, with both parameters constrained to be positive based on physical principles. The optimization was performed using the Levenberg–Marquardt algorithm, ensuring a robust fit to the experimental data.

**Figure 3:**
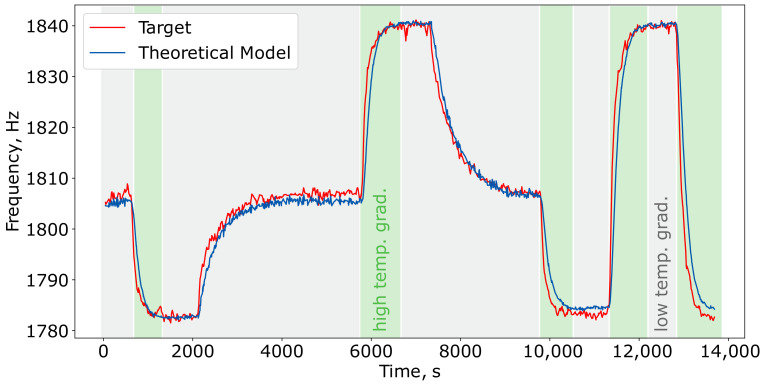
Results of the simple theoretical model (9) applied to the test subset of the “1010 ppm ${\mathrm{CH}}_{4}$” dataset.

The primary metrics for fitting this model to the data from the “1010 ppm ${\mathrm{CH}}_{4}$” dataset are summarized in Table 2. The application of the trained theoretical model to the test subset is illustrated in Figure 3. In this figure, the areas with gray and green shading indicate time intervals characterized by low- and high-temperature gradients, respectively.

**Figure 4:**
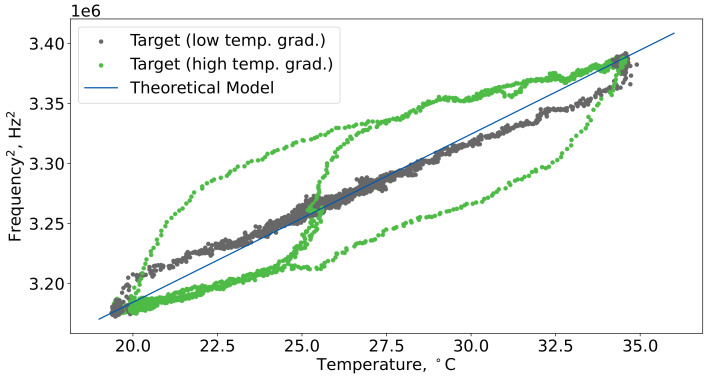
Data from the “1010 ppm ${\mathrm{CH}}_{4}$” dataset plotted in terms of $T_{t}$ and ${\left(f_{t}^{r}\right)}^{2}$, along with the fitting curve derived from the theoretical model (9). The gray areas indicate sections with low-temperature gradients, while the green areas represent regions with high-temperature gradients.

Figure 4 illustrates the relationship between the predicted resonance frequency (blue line) and the target resonance frequency (green and gray dots) as a function of temperature. The gray dots indicate measurements taken in regions with low-temperature gradients, while the green dots represent measurements from areas with high-temperature gradients (the same as in Figure 3). The figure demonstrates that the basic theoretical model reasonably and accurately captured the cell’s resonance frequency in low-temperature gradient regions. However, it struggled to provide accurate predictions in regions characterized by high-temperature gradients.

### 5.2. LSTM-Based Model

To estimate the real-time gas temperature without delay, we used a single-layer long short-term memory (LSTM) neural network. LSTM, a recurrent neural network architecture introduced by Hochreiter and Schmidhuber in 1997 [26], was selected due to its effectiveness in capturing time dependencies and handling sequential data. This architecture is particularly suitable for our problem, as it is capable of approximating solutions to the differential equation of thermal conductivity (8), which governs the temperature distribution and dynamics in the system.

It is widely known that LSTM architectures can be used to solve various types of differential equations. In [27], the authors applied LSTM networks to solve the linear advection equation, which involves the propagation of the initial conditions at a constant speed; the inviscid Burgers equation, which generates shockwaves; and the chaotic Kuramoto–Sivashinsky equation. Similarly, in [28], LSTM was employed to solve the Burgers equation, the λ-ω reaction-diffusion equation, and the Gray–Scott equation. In [29], the LSTM architecture was used to solve the generalized nonlinear Schrödinger equation. Furthermore, the application of LSTM to heat equations has been explored in other works, such as [30,31,32], showing the versatility of LSTM networks in handling complex physical models.

**Figure 5:**
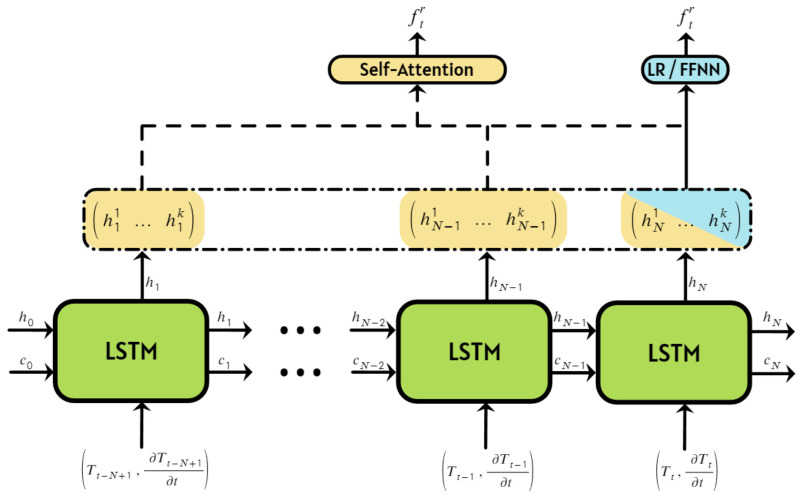
Neural network architecture featuring an LSTM block for time-series data processing.

**Figure A1:**
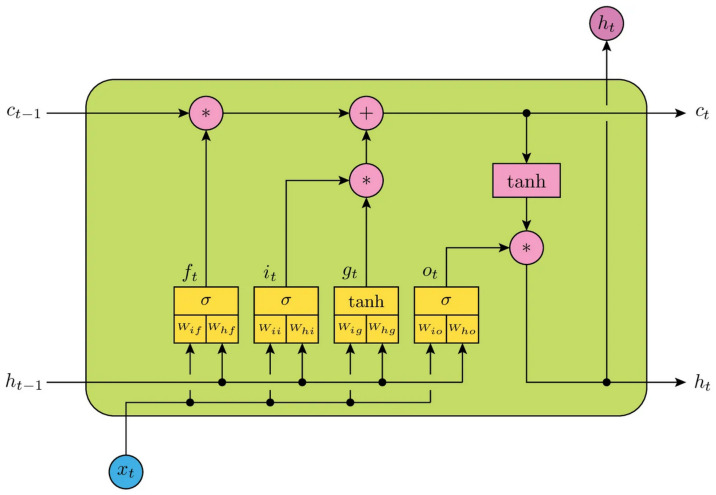
Architecture of LSTM block in PyTorch [33].

The architecture of the recurrent neural network used in this study is shown in Figure 5. We used the LSTM block architecture implemented in PyTorch (Figure A1), a detailed description of which can be found in [33] and Appendix B.

The LSTM network was fed with two time-series inputs to predict the resonance frequency $f_{t}^{r}$ at time *t*: the temperature series $\left(T_{t-N+1},\ldots ,T_{t}\right)$ and the temperature derivative series $\left(\frac{\partial T_{t-N+1}}{\partial t},\ldots ,\frac{\partial T_{t}}{\partial t}\right)$. Thus, at each time step *t*, the LSTM cell received two values: $T_{t}$ and $\frac{\partial T_{t}}{\partial t}$. The hyperparameter *N* was an adjustable parameter of the LSTM network. By using both the current temperature and its derivative, the model was able to compensate for potential temperature estimation errors and account for any lag in determining the actual gas temperature. Incorporating values from previous time steps allowed the LSTM network to more accurately solve the differential equation and predict the resonance frequency in real time.

The output of the *n*-th LSTM cell was a vector $h_{n}=\left(h_{n}^{1},\ldots ,h_{n}^{k}\right)$ of length *k* (hidden size), n=1,⋯,N. The hidden size, *k*, was another adjustable parameter of the LSTM network, controlling the dimensionality of the output from each cell. The entire output of the LSTM layer was represented as a matrix $H={\left(h_{1},\ldots ,h_{N}\right)}^{T}$ with dimensions (N×k). The outputs from the LSTM cells (either all or just the final one) were then passed into one of three different NN architectures (Figure 5), which are discussed in detail later.

Hyperparameter tuning for these architectures was performed using the open-source Optuna library, utilizing the HyperbandPruner [34] and the TPESampler [35] algorithms. In addition to the hidden size (*k*) and the input sequence length (*N*), we optimized the batch size, learning rate scheduler parameters, and other architecture-specific parameters. A detailed overview of all tuned parameters can be found in Appendix D. The optimal hyperparameters were identified after conducting 400 trials. The neural networks were trained for 200 epochs using a GIGABYTE GeForce RTX 4090 WINDFORCE graphics card. The models were trained on the “all ${\mathrm{CH}}_{4}$ conc.” dataset (see Table 1).

#### 5.2.1. Linear Regression

As a baseline architecture, we considered a simple linear regression layer at the output of the LSTM. The input data consists of the outputs from the last LSTM cell $\left(h_{N}^{1},\ldots ,h_{N}^{k}\right)$, where k=3 and N=4. The resonance frequency was then calculated using the following formula: $f_{t}^{r}=\beta_{0}+\beta_{1}h_{N}^{1}+\ldots +\beta_{k}h_{N}^{k}$, where $\beta_{0},\ldots ,\beta_{k}$ are the trainable weights of the model. Both the LSTM and the linear regression layer (LSTM+LR) were trained simultaneously, resulting in a model with a total of 88 internal parameters (including those of the LSTM). The Mean Squared Error (MSE) was used as the loss function, while the Adam optimizer facilitated the optimization process. Additionally, the learning rate was reduced when the validation loss reached a plateau.

#### 5.2.2. Fully Connected Neural Network

As an alternative approach, we implemented a fully connected feed-forward neural network (FFNN) following the LSTM layer. The output from the final LSTM cell served as input to this network, which consisted of two hidden layers. The number of neurons in each hidden layer, denoted as *n*, was optimized to either 2 or 3. The final output of the network was the predicted resonance frequency of the gas cell, $f_{t}^{r}$. To enhance performance, the activation function for the fully connected layers was chosen to be LogSigmoid. The architecture of the fully connected FFNN head is detailed in Table 3. The resulting model, referred to as LSTM + FFNN, contained a total of 263 internal parameters, including those from the LSTM layer. Training was conducted using the Adam optimization algorithm, and the learning rate was adjusted downward when the validation loss reached a plateau, similar to the training process employed for the NN with the linear regression layer.

#### 5.2.3. Self-Attention Neural Network

**Figure A2:**
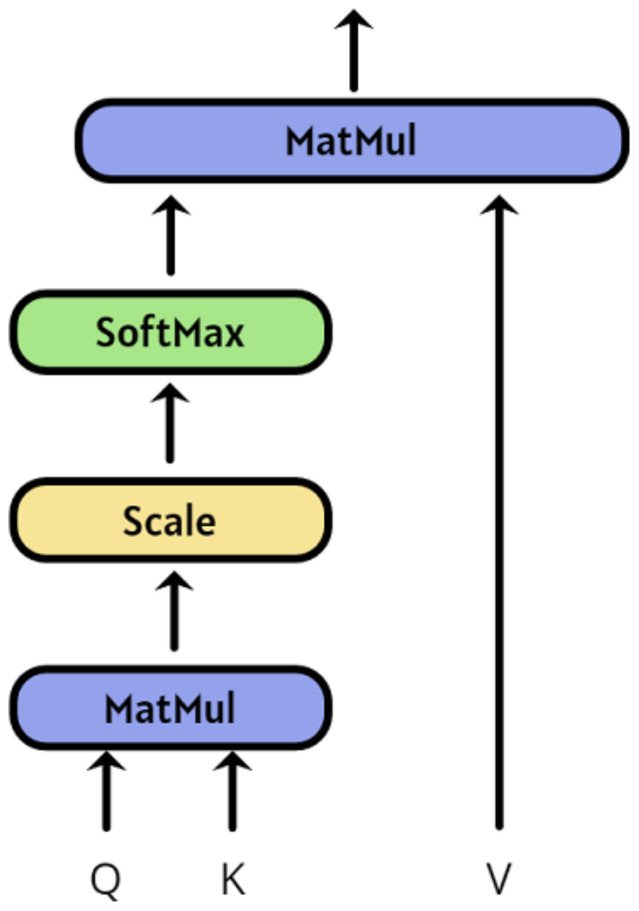
The architecture of the scaled dot-product attention block.

The self-attention (SA) mechanism, often referred to as scaled dot-product attention, is a key element of the Transformer architecture and has demonstrated significant success across various domains, particularly natural language processing [21]. The structure of the scaled dot-product attention block is illustrated in Figure A2 and further explained in Appendix C. Self-attention enables the model to assign varying levels of importance to different parts of the input sequence when creating its internal representation, enhancing the model’s ability to capture complex dependencies within the data.

In our approach, we applied the self-attention mechanism to the output of the LSTM block, which consists of a matrix $H={\left(h_{1},\ldots ,h_{N}\right)}^{T}$ with dimensions (N×k). Here, $h_{n}=\left(h_{n}^{1},\cdots ,h_{n}^{k}\right)$ is the output from the *n*-th LSTM cell, where n=1,⋯,N, and *k* is the hidden size of the LSTM (as described in Section 5.2). This matrix acts as the input embedding for the self-attention mechanism.

After the self-attention block, we used a fully connected neural network with a single hidden layer. This network’s primary function was to predict the resonance frequency at a given time using the outputs from the self-attention block. The full architecture of this model is presented in Table 4. We utilized the AdamW optimization algorithm in conjunction with the NoamLR scheduler to train the self-attention-based neural network, ensuring efficient convergence and adaptive learning rate adjustments [21].

## 6. Results and Discussion

Table 5 compares the testing metrics for all models. The models were trained on the “all ${\mathrm{CH}}_{4}$ conc.” dataset, and the metrics were derived from separate test subsets corresponding to each concentration (i.e., 15% of the original dataset, with approximately 1000 points in each test subset). All LSTM-based models demonstrated strong performance, with mean absolute error (MAE) values below 1 Hz for all concentrations, except for 97 ppm. The LSTM + SA model generally delivered the best or nearly the best performance across most concentrations, especially at the more challenging concentration of 97 ppm. However, the differences between the models were generally small, and all models performed well across the majority of concentrations, particularly when an MAE < 1 Hz was considered an acceptable result. The 97 ppm concentration appeared to be the most difficult, as all models exceeded the 1 Hz threshold in this case.

In our experimental analysis, the LSTM + SA model achieved the lowest MAE, ranging from 0.74 to 1.08 Hz. This represents a more than twofold improvement over the theoretical model for predicting the resonant frequency, which exhibited an MAE between 2.07 and 2.58 Hz.

**Figure 6:**
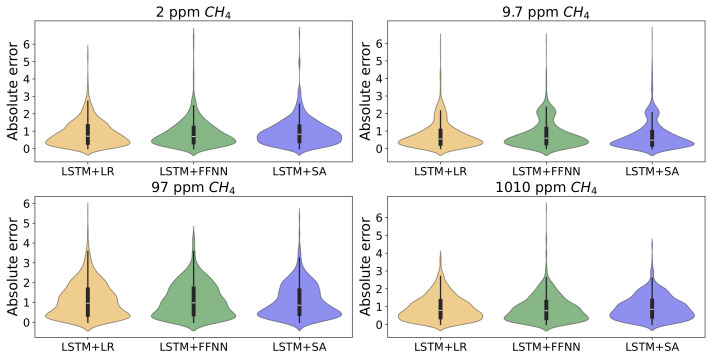
Violin plots illustrating the distribution of the mean absolute error for all models across each ${\mathrm{CH}}_{4}$ concentration.

For a more detailed comparison of the LSTM-based models, we analyzed the MAE distribution using violin plots, as shown in Figure 6. The shapes of these distributions demonstrate that all models performed effectively, with the median MAE staying below 1 Hz for most concentrations. However, for the 97 ppm ${\mathrm{CH}}_{4}$ concentration, the LSTM + SA model stood out with a notably lower median MAE, well below 1 Hz. Additionally, the distributions’ tails decayed faster as the MAE increased, indicating fewer extreme errors for this challenging concentration.

**Figure 7:**
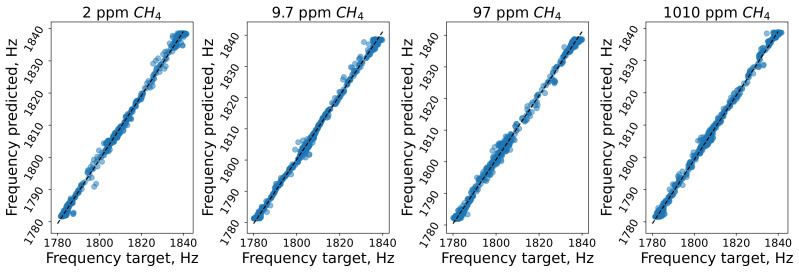
Scatter plots representing the correlation between the predicted and target resonance frequencies for the model with the LSTM+SA architecture for all concentrations of ${\mathrm{CH}}_{4}$.

The error distribution across the range of cell resonance frequencies for the LSTM + SA model is shown in Figure 7. The spread of points around the diagonal is relatively consistent, without significant deviations or outliers for any specific concentration. This suggests that the model performed consistently across different frequencies and methane concentrations.

**Figure 8:**
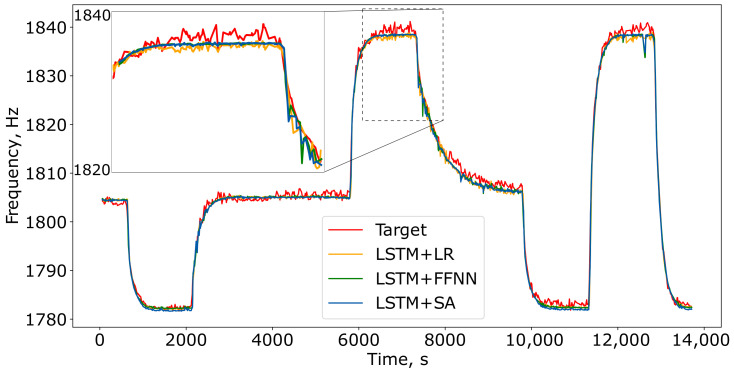
The result of applying the best-trained models to the test subset of the “2 ppm ${\mathrm{CH}}_{4}$” dataset. The performance metrics for these models are shown in Table 5.

Figure 8 illustrates how the best-trained models reconstructed the resonance frequency on the test subset of the “2 ppm ${\mathrm{CH}}_{4}$” dataset. All models effectively captured the frequency variations, with the LSTM+SA model providing the smoothest and most accurate predictions over the 4-hour measurement period.

The proposed approach based on LSTM networks enables real-time resonance frequency tracking, which significantly improves the accuracy of gas concentration measurements. In our previous study [17], implementing the extremum-seeking control algorithm for frequency tracking while measuring the concentration of methane impurities in the air over a 2-minute period resulted in a 15% reduction in the measurement error compared to measurements conducted without frequency tracking.

A promising direction for future research involves developing algorithms that incorporate both temperature sensor readings and the measured concentration of the target gas to enhance resonance frequency estimation at higher gas concentrations. Since the resonance frequency of the cell is influenced by the gas concentration, future models should use both temperature and concentration data as inputs. This integrated approach would provide more precise resonance frequency predictions, particularly under high gas concentration conditions, further improving the sensor’s accuracy and reliability in various operational settings.

## 7. Conclusions

We theoretically and experimentally investigated the impact of temperature fluctuations and varying gas concentrations on shifts in the PAD resonance frequency. To address the challenges caused by changing cell temperatures, we proposed a novel approach: predicting the resonance frequency of the gas cell using temperature data. Our experimental dataset comprised multiple measurements, capturing both the temperature and resonance frequency across different gas mixtures during repeated heating and cooling cycles of the PAD, which operated under various temperature gradients.

We developed predictive models using LSTM networks and self-attention mechanisms to estimate the resonance frequency from the recorded temperature data. After training, these models achieved real-time predictions with a mean absolute error below 1 Hz for frequency shifts exceeding 30 Hz over a four-hour period. This approach enables continuous tracking of the resonance frequency without interrupting laser operation, significantly enhancing the accuracy of gas concentration measurements and supporting the long-term stability of photoacoustic gas sensors.

## Figures and Tables

**Table 1:** Overview of the constructed datasets.

Dataset	Number of Points			Range of	
	**Train**	**Validation**	**Test**	**$T_{t}$, °C**	**$f_{t}^{r}$, Hz**
2 ppm ${\mathrm{CH}}_{4}$	4663	999	1000	19.5÷34.4	1781.4÷1841.8
9.7 ppm ${\mathrm{CH}}_{4}$	4548	975	975	19.1÷34.7	1779.9÷1839.9
97 ppm ${\mathrm{CH}}_{4}$	4604	987	987	19.6÷34.8	1780.0÷1840.1
1010 ppm ${\mathrm{CH}}_{4}$	4656	998	998	19.4÷34.9	1781.2÷1841.7
all ${\mathrm{CH}}_{4}$ conc.	4617	989	990	19.2÷34.7	1779.9÷1841.7
all ${\mathrm{CH}}_{4}$ conc. no discard	18,471	3959	3960	19.1÷34.9	1779.9÷1841.8

**Table 2:** Metrics of the theoretical model for all the subsets of the “1010 ppm ${\mathrm{CH}}_{4}$” dataset.

Model	Train		Validation		Test	
	**MSE, Hz^2^**	**MAE, Hz**	**MSE, Hz^2^**	**MAE, Hz**	**MSE, Hz^2^**	**MAE, Hz**
Theoretical (9)	12.02	1.97	14.40	2.14	11.50	1.97

**Table 3:** Architecture of the fully connected FFNN head block with optimal values of the hyperparameters: *k*—hidden size; *n*—number of neurons (see Appendix D for details).

Block	Name of Layer	Input Shape	Output Shape	Num. of Parameters
FC 0	Fully Connected	k=6	n=2	14
	Activation (LogSigmoid)	n=2	n=2	–
FC 1	Fully Connected	n=2	n=2	6
	Activation (LogSigmoid)	n=2	n=2	–
FC 2	Fully Connected	n=2	1	3

**Table 4:** Architecture of the self-attention with FFNN head block with optimal values of the hyperparameters: *k*—hidden size; *n*—number of neurons (see Appendix D for details).

Block	Name of Layer	Input Shape	Output Shape	Num. of Param.
SA	Self-Attention	N×k(7×7)	$N\times d_{model}\;\left(7\times 3\right)$	63
	Flatten	$N\times d_{model}\;\left(7\times 3\right)$	$Nd_{model}=21$	–
FC 0	Fully Connected	$Nd_{model}=21$	3	66
	Activation (Sigmoid)	3	3	–
FC 1	Fully Connected	3	1	4

**Table 5:** Metrics of all models, calculated on the test subsets corresponding to each ${\mathrm{CH}}_{4}$ concentration.

${\mathrm{CH}}_{4}$ Conc.	Model	Test	
		**MSE, ${\mathrm{Hz}}^{2}$**	**MAE, Hz**
2 ppm	LSTM + LR	1.33	0.89
	LSTM + FFNN	1.39	0.88
	LSTM + SA	1.47	0.95
	Theoretical (9)	12.85	2.07
9.7 ppm	LSTM + LR	1.19	0.78
	LSTM + FFNN	1.30	0.84
	LSTM + SA	1.15	0.74
	Theoretical (9)	16.64	2.58
97 ppm	LSTM + LR	2.00	1.12
	LSTM + FFNN	1.96	1.12
	LSTM + SA	1.85	1.08
	Theoretical (9)	12.70	2.22
1010 ppm	LSTM + LR	1.34	0.93
	LSTM + FFNN	1.35	0.91
	LSTM + SA	1.34	0.95
	Theoretical (9)	12.02	2.12

## Data Availability

Dataset available on request from the authors.

## Appendix A. Theoretical Expression for the Resonance Frequency Shift in a Gas-Filled Acoustic Cell

The ratio of resonance frequencies, as the gas parameters change from $\left(T_{0},{\vec{n}}_{0}\right)$ to $\left(T,\vec{n}\right)$, can be calculated using Equations (2) and (4) as follows:

(A1) $$\frac{f^{r}\left(T,\vec{n}\right)}{f^{r}\left(T_{0},{\vec{n}}_{0}\right)}=\sqrt{\frac{\gamma (T,\vec{n})}{\gamma (T_{0},{\vec{n}}_{0})}}\cdot \sqrt{\frac{M({\vec{n}}_{0})}{M(\vec{n})}}\cdot \sqrt{\frac{T}{T_{0}}}\cdot \frac{\kappa (T,\vec{n})}{\kappa (T_{0},{\vec{n}}_{0})},$$

where the adiabatic index $\gamma (T,\vec{n})$ and the molar mass $M(\vec{n})$ of the mixture are calculated according to Formulas (6) and (7), respectively. The relative deviation of the resonance frequency, based on relation (A1), is defined as

(A2) $$\left(\frac{\Delta f^{r}}{f^{r}}\right){|}_{\left(T_{0},{\vec{n}}_{0}\right)}=\left(\frac{\partial \ln(f^{r})}{\partial T}\right){|}_{\left(T_{0},{\vec{n}}_{0}\right)}\Delta T+\left(\frac{\partial \ln(f^{r})}{\partial \vec{n}}\right){|}_{\left(T_{0},{\vec{n}}_{0}\right)}^{\sum \delta n_{i}=0}\cdot \Delta \vec{n},$$

with the temperature and concentration corrections, using (A1), calculated as follows:

(A3) $$\left(\frac{\partial \ln(f^{r})}{\partial T}\right){|}_{\left(T_{0},{\vec{n}}_{0}\right)}=\frac{1}{2T_{0}}+\frac{1}{2}\frac{\partial \ln(\gamma )}{\partial T}{|}_{\left(T_{0},{\vec{n}}_{0}\right)}+\frac{\partial \ln(\kappa )}{\partial T}{|}_{\left(T_{0},{\vec{n}}_{0}\right)},$$

(A4) $$\left(\frac{\partial \ln(f^{r})}{\partial \vec{n}}\right){|}_{\left(T_{0},{\vec{n}}_{0}\right)}^{\sum \delta n_{i}=0}=\frac{1}{2}\frac{\partial \ln(\gamma )}{\partial \vec{n}}{|}_{\left(T_{0},{\vec{n}}_{0}\right)}^{\sum \delta n_{i}=0}-\frac{{\vec{m}}^{T}}{2M({\vec{n}}_{0})}+\frac{\partial \ln(\kappa )}{\partial \vec{n}}{|}_{\left(T_{0},{\vec{n}}_{0}\right)}^{\sum \delta n_{i}=0}.$$

In the temperature correction (expression (A3)), the second term can be neglected compared to the first term because the ratio of the second term to the first term is insignificantly small, and accounting for the second term provides a correction to the change in the resonance frequency $f^{r}$ only in the second decimal place (see Table A1). In Table A1, the reference data [36] for various gases are presented, including the molar mass and adiabatic index at different temperatures, as well as the ratio of the second term to the first term in the resonance frequency correction for temperature (Equation (A3)) at a temperature of $T_{0}=283\;K$.

**Table A1:** Reference data on the molar mass and adiabatic index at different temperatures for various gases, as well as the ratio of the second term to the first term in the resonance frequency correction for temperature (Equation (A3)).

Gas	M, g/mol	γ (0 °C)	γ (10 °C)	γ (25 °C)	$T_{0}\cdot \frac{\partial \ln(\gamma )}{\partial T}{|}_{T_{0}=283K}$
$N_{2}$	28.01	1.4019	1.4015	1.4013	−0.0048
${\mathrm{CH}}_{4}$	16.04	1.3164	1.3104	1.3062	−0.0881
${\mathrm{SF}}_{6}$	146.06	1.1074	1.1017	1.0984	−0.0924

To calculate the shift in the resonance frequency using Formula (A2), it is necessary to know the value of the resonance frequency at a given temperature and the concentrations of the mixture components. The resonance frequency can be calculated using Formula (4), for which it is necessary to estimate the speed of sound ${\hat{c}}_{s}$ and the ratio $\frac{\kappa }{L}$. Table A2 presents the theoretical values of the speed of sound ${\hat{c}}_{s}$, calculated using (2), (6), (7), and the experimental data on the cell resonance frequency $f^{r}$ at different concentrations of the gas ${\mathrm{SF}}_{6}$ in a nitrogen ($N_{2}$) atmosphere at a constant temperature [37]. These data allow us to estimate the value $f^{r}/{\hat{c}}_{s}\approx \kappa /L$ (see Formula (4)), the values of which are also presented in Table A2.

**Table A2:** Theoretical values of the speed of sound and experimental values of the resonant frequency of the cell at different concentrations of ${\mathrm{SF}}_{6}$ in a $N_{2}$ atmosphere [37].

Impurity	Impurity Conc., %	Temp., *T*, K	Theor. ${\hat{c}}_{s}$, m/s	Exper. $f^{r}$, Hz	$f^{r}/{\hat{c}}_{s}$, m^−1^
${\mathrm{SF}}_{6}$	–	296.5	351.25	1782	5.073
	0.1	296.7	350.42	1777	5.071
	1	296.8	342.72	1736	5.065
	10	296.9	284.68	1438	5.051
	100	297.0	136.29	688	5.048

The last column of Table A2 shows that the ratio κ/L changes insignificantly across the entire range of considered concentrations of ${\mathrm{SF}}_{6}$ in a $N_{2}$ atmosphere, allowing us to consider the value κ/L≈const in the future. Based on the data on the molar mass of gases in Table A1, assuming κ/L≈5.073 (pure nitrogen) and using Formulas (2), (6), (7), and (4), it is possible to calculate the theoretical values of the resonance frequency $f^{r}$ for a mixture of ${\mathrm{SF}}_{6}$ and ${\mathrm{CH}}_{4}$ at different concentrations in a nitrogen atmosphere at a constant temperature. The obtained theoretical values of the resonance frequency for various mixtures of the considered gases are given in Table A3. For the calculation of the resonance frequency, the temperature values for the ${\mathrm{SF}}_{6}$ mixtures were taken from Table A2, while for the ${\mathrm{CH}}_{4}$ mixtures, the temperature was assumed to be 296.5 Kelvin (the same as for pure nitrogen, as shown in Table A2).

Table A3 also presents the theoretical calculations of the change in the resonance frequency $\Delta f^{r}$ of the gas cell, depending on the gas mixture and its temperature, for a temperature change of 1 Kelvin and/or a change in the concentration of the mixture components by 1 ppm. These calculations are obligatory for determining which of these two factors (see expression (A2)) is the main cause of the change in the cell resonance frequency. Since the gas mixtures considered in this table are two-component, the change in the concentration of the mixture components is −1 ppm for the atmospheric gas (nitrogen) and +1 ppm for the impurity gas (either ${\mathrm{SF}}_{6}$ or ${\mathrm{CH}}_{4}$). For an ideal gas, κ≡1 and expressions (A3) and (A4) are simplified accordingly to

(A5) $$\left(\frac{\partial \ln(f^{r})}{\partial T}\right){|}_{\left(T_{0},{\vec{n}}_{0}\right)}=\frac{1}{2T_{0}},$$

(A6) $$\left(\frac{\partial \ln(f^{r})}{\partial \vec{n}}\right){|}_{\left(T_{0},{\vec{n}}_{0}\right)}=\frac{1}{2}\frac{\partial \ln(\gamma )}{\partial \vec{n}}{|}_{\left(T_{0},{\vec{n}}_{0}\right)}-\frac{{\vec{m}}^{T}}{2M({\vec{n}}_{0})}.$$

The calculation of the change in the resonance frequency $\Delta f^{r}$ was performed using Formula (A2), taking into account expressions (A5) and (A6).

From Table A3, it follows that the determination of the resonance frequency for methane (${\mathrm{CH}}_{4}$) and sulfur hexafluoride (${\mathrm{SF}}_{6}$) in a pure nitrogen atmosphere differs. By comparing the ratios $\Delta f_{\mathrm{ppm}}^{r}/\Delta f_{K}^{r}$ for ${\mathrm{CH}}_{4}$ and ${\mathrm{SF}}_{6}$, it can be deduced that the shift in the resonance frequency due to the change in the concentration of sulfur hexafluoride is about 10 times more pronounced than the shift in the resonance frequency due to the change in the concentration of methane. The precise determination of the resonance frequency and concentration of ${\mathrm{SF}}_{6}$ is a more complex task compared to methane.

Thus, when determining the concentration of methane below 1% (10,000 ppm), it can be assumed that the entire shift in the resonance frequency is due to a change in the gas temperature. For sulfur hexafluoride, this statement is valid only for concentrations below 0.1% (1000 ppm).

**Table A3:** Theoretical calculations of the change in the resonance frequency.

Impurity	Impurity Conc., %	Temp. *T*, K	Theor. $f^{r}$, Hz	$\Delta f_{\mathrm{ppm}}^{r}$, $10^{-4}$ Hz/ppm	$\Delta f_{K}^{r}$, Hz/K	$\Delta f_{\mathrm{ppm}}^{r}/\Delta f_{K}^{r}$, $10^{-4}$ K/ppm
${\mathrm{SF}}_{6}$	–	296.5	1781.89	−45.4	3.0	−15.1
	0.1	296.7	1777.68	−45.1	3.0	−15.1
	1	296.8	1738.61	−42.4	2.9	−14.5
	10	296.9	1444.18	−25.4	2.4	−10.5
	100	297.0	691.40	−3.0	1.7	−2.6
${\mathrm{CH}}_{4}$	–	296.5	1781.89	3.0	3.0	1.0
	0.01	296.5	1781.92	3.0	3.0	1.0
	0.1	296.5	1782.19	3.0	3.0	1.0
	1	296.5	1784.92	3.0	3.0	1.0
	10	296.5	1813.40	3.3	3.1	1.1
	100	296.5	2273.75	7.9	3.8	2.1

## Appendix B. Architecture of LSTM Block

Figure A1 shows the architecture of the LSTM block, where $x_{t}$ is the external input at time *t* and $h_{t-1}$ and $h_{t}$ are the hidden states at times t−1 (“input”) or t (“output”). Despite its name, the hidden state is also used as the output or input for the next layer of LSTM cells (for multi-layer networks). $c_{t-1}$ and $c_{t}$ are the “cell state” or “memory” at times t−1 and *t*, respectively, and $f_{t}$ is the result of the forget gate. For values close to zero, the cell will “forget” its memories $c_{t-1}$ from the past; for values close to one, it will remember its history. $i_{t}$ is the result of the input gate, determining how important the (transformed) new external input is; $g_{t}$ is the result of the cell gate, a non-linear transformation of the new external input $x_{t}$; and $o_{t}$ is the result of the output gate, which controls how much of the new cell state $c_{t}$ should go to the output (and the hidden state).

The operating algorithm of the LSTM block is presented in Algorithm A1.

**Algorithm A1:** LSTM Algorithm.
**Functions:** $i_{t}$—input gate; $f_{t}$—forget gate; $c_{t}$—cell gate; $o_{t}$—output gate; σ—sigmoid function; ⊙—Hadamard product.
**Require:** Input $x_{t}$ at the time *t*, hidden state $h_{t-1}$ of the layer at time t−1, cell state $c_{t-1}$ at time t−1.
$i_{t}\leftarrow \sigma \left(W_{ii}x_{t}+b_{ii}+W_{hi}h_{t-1}+b_{hi}\right)$
$f_{t}\leftarrow \sigma \left(W_{if}x_{t}+b_{if}+W_{hf}h_{t-1}+b_{hf}\right)$
$g_{t}\leftarrow tanh\left(W_{ig}x_{t}+b_{ig}+W_{hg}h_{t-1}+b_{hg}\right)$
$o_{t}\leftarrow \sigma \left(W_{io}x_{t}+b_{io}+W_{ho}h_{t-1}+b_{ho}\right)$
$c_{t}\leftarrow f_{t}⊙c_{t-1}+i_{t}⊙g_{t}$
$h_{t}\leftarrow o_{t}⊙tanh\left(c_{t}\right)$

## Appendix C. Architecture of the Self-Attention Block

Figure A2 shows the architecture of the scaled dot-product attention block. Self-attention allows a model to weigh the importance of different parts of the input sequence when constructing a representation of the sequence.

The input to the self-attention mechanism is a set of embeddings, typically representing words or tokens in a sequence. Each embedding is a vector that captures the features of the corresponding word or token. In our case, the representation obtained after the LSTM block is used as the input embedding to the self-attention mechanism, namely the matrix $H={\left(h_{1},\ldots ,h_{N}\right)}^{T}$ with dimensions (N×k), where $h_{n}=\left(h_{n}^{1},\cdots ,h_{n}^{k}\right)$ is the output of the *n*-th LSTM cell; n=1,⋯,N; and *k* is the hidden size (see Section 5.2). For each input embedding, three new vectors are computed: the query (Q), the key (K), and the value (V). These vectors are obtained by multiplying the input embedding by three different learned weight matrices $W_{Q}$, $W_{K}$, and $W_{V}$:

(A7) $$Q=HW_{Q},\;K=HW_{K},\;V=HW_{V},$$

where the dimensions of the matrices $W_{Q},\;W_{K},$ and $\;W_{V}$ are $\left(k\times d_{model}\right)$, and the dimensions of the new vectors Q,K, and *V* are $\left(N\times d_{model}\right)$. The value $d_{model}$ is a hyperparameter that can be adjusted in the range from 1 to 3.

The relevance of each element in the sequence to another is calculated by taking the scalar product of the corresponding query and key vectors. This produces a score that indicates how much focus should be given to each element in the sequence: $score\left(Q_{i},K_{j}\right)=Q_{i}\cdot K_{j}$. To prevent large values resulting from the dot product, which can lead to vanishing gradients, the scores are scaled by the square root of the dimension of the key vectors, d: $scaled\_score\left(Q_{i},K_{j}\right)=Q_{i}\cdot K_{j}/\sqrt{d_{model}}$.

The scaled scores are then passed through a softmax function to obtain attention weights. These weights determine the importance of each element in the sequence relative to the others: $\alpha_{ij}=\mathrm{softmax}\left(Q_{i}\cdot K_{j}/\sqrt{d_{model}}\right)$.

Each element’s representation is updated by taking a weighted sum of the value vectors, where the weights are the attention scores obtained from the softmax function:

(A8) $$Attention\left(Q,K,V\right)=\mathrm{softmax}\left(\frac{Q\cdot K^{T}}{\sqrt{d_{model}}}\right)V,$$

where the output of Attention(Q,K,V) is a matrix of size $\left(N\times d_{model}\right)$. We then apply flattening to the given matrix to transform it into a vector of length $Nd_{model}$. Next, we use a fully connected network with one hidden layer, the purpose of which is to predict the resonance frequency at a given moment. The complete architecture of this approach can be seen in Table 4.

## Appendix D. Description of Hyperparameters

The hyperparameters for the three models (LSTM + LR, LSTM + FFNN, and LSTM + SA) were optimized. Table A4 and Table A5 present the ranges of the hyperparameter values, while Table A6 provides the corresponding optimal values.

**Table A4:** Tunable hyperparameters for the LSTM block, common to all the architectures.

Hidden Size (*k*)	Input Sequence Length (*N*)
2÷7	3÷8

**Table A5:** Tunable hyperparameters for the LR, FFNN, and SA architectures.

Architecture		LR	FFNN	SA
Hyperparameter	batch size	8÷256	4÷16	4÷16
	learning rate	$10^{-3}÷10^{-1}$	$10^{-3}÷10^{-1}$	$10^{-3}÷1$
	scheduler reduction factor	0.1÷0.9	0.2÷0.9	–
	number of neurons	–	2÷3	3
	warmup steps	–	–	5÷30
	$d_{model}$	–	–	1÷3
	activation		ReLU, Sigmoid,	ReLU, Sigmoid,
		–	SiLU, Hardshrink,	SiLU, Hardshrink,
			Tanh, LogSigmoid	Tanh, LogSigmoid

**Table A6:** Optimal values of the hyperparameters for all the architectures.

Architecture		LR	FFNN	SA
Hyperparameter	LSTM hidden size (*k*)	3	6	7
	LSTM input seq. len. (*N*)	4	8	7
	batch size	8	8	8
	learning rate	0.040	0.064	0.163
	scheduler reduction factor	0.802	0.768	–
	number of neurons	–	2	3
	warmup steps	–	–	6
	$d_{model}$	–	–	3
	activation	–	LogSigmoid	Sigmoid
